# 

**Published:** 1800-04

**Authors:** 


### NEW MEDICAL PUBLICATIONS IN MARCH.

Annals of Medicine for the year 1799; by Andrew Duncan, Sen. M. D. and Andrew Duncan, Jun. M. D. 8s. bds. Robinſons.

The Efficacy of Perkins's Metallic Tractors, exemplified by a number of Caſes on the Human Body and Horſes, from the firſt literary Characters. With a Diſcourſe, in which the Attempts of Dr. Haygarth to detract from the Merits of the Tractors, are fully confuted. By B. D. Perkins, A. M. 1s. Johnſon.

A brief Hiſtory of Epidemic and Peſtilential Diſeaſes, with the principal Phenomena of the Phyſical World, which precede and accompany them; and Obſervations deduced from the Facts ſtated; by Noah Webster, 2 vol. 8vo. 18s. boards. Robinſons.

Inſtitutions of the Practice of Medicine, by Jo. Baptist Burserius de Kanifeld. Tranſlated from the Latin by Wm. Cullen Brown. (5 vol. 8vo.) Vol. I. 8s. boards. Robinſons.

### NEW MEDICAL PUBLICATIONS IN FRANCE.

*Diſſertation, &c.* An Anatomico-chirurgical Diſſertation on Fractures of the Neck of the Femur. By A. Richerand, M. D. Member of the Philomathic Society at Paris, &c. &c. Paris, Meguignon.

*Bibliographie Analytique de Medicine, &c.*—Analytico-Medical Library; or a Journal compiled from the beſt new works in Latin or French, on the ſubjects of Clinical and Prophylactic Medicine and Hygiene. By Laurent Bodin. Two numbers of this work are publiſhed, forming together two ſheets in 12mo.

*Liſte Chronologique, &c.*—A Chronological Liſt of the Works of the Phyſicians and Surgeons of Bourdeaux, and of thoſe who have practiſed the Curative Art in that City; with Annotations, and an Elegy on Pierre Default, M. D. By J. Tournon, M. D. Member of the Medical Society at Bourdeaux, &c. a Pamphlet of 47 pages, 8vo. price 1 franc, 50 centimes; publiſhed at Bourdeaux by F. Pellier Lavalle.

*Revolution de la Medicine*, &c.—Revolution in the Science of Medicine, or the regeneration of the Curative Art; containing a new new doctrine on animal organisation and disorganisation, preferable to the ancient systems, and shewing the necessity of a reformation in several essential points of theory and practice. By Citizen LEBESCHU, M. D. 2 vols. Paris, Méguignon.

*Experiences sur le Galvanisme* &c.—Experiments on Galvanism, and on the general irritation of the muscular and nervous fibres: Translated from the German of Frederic Alexander Humboldt: By J. F. N. JADELOT, M. D. In one volume 8vo. 600 pp. price 6 francs. Paris. Fuchs.

*Observation*, &c.—Observations on the Cæsarean Operation, successfully performed, with the description of a new method of performing it: By Cit. JAQUES ANDRE MILLOT, Manmidwife. 38 pp. 8vo. Paris, Croullebois.

*Tableau Methodique*, &c.—Methodical plan of a course of Medical Natural History; in which are combined and classed the principal mineral waters of the Republic; the places from which they proceed are likewise stated, as also their temperature, the substances they contain, their virtues, uses, &c. &c. which have never yet been given in any medical work. By BERNARD PEYVILHE, Professor of Medical Natural History in the School of Medicine at Paris. 1 vol. 8vo. near 600 pp. price 7 francs in boards. Paris. Widow Panckoucke.

*Instruction*, &c.—Rules for the practice of inoculation for the small-pox; to which is added, an Essay on the nature and treatment of that disorder, extracted from the Lectures of Cit. PORTAL, Professor of Medicine at the College of France: By Cit. SALMADE, M. D. Senior Surgeon at the National Hospital for Invalids, &c. &c. Paris. Merlin.

[ *The List of New German Publications in our next.* ]

*** On account of the numerous new Articles in the present Retrospect, the conclusion of "*Prof. Göttling's Manual of the Theory and Practice of Chemistry*," from p. 189 of Number XII. and "*Prof. Schmid's Philosophic System of Physiology*," from p. 283 of our last, shall be concluded in the next Number.

### To CORRESPONDENTS.

*We have received Communications from Dr. Vage; Mr. Dunning, Mr. Wagstaffe, Mr. Blackburn, Mr. Lipscomb, R. H. &c. which shall be duly attended to.*

